# Design and Application of Toxic and Harmful Gas Monitoring System in Fire Fighting

**DOI:** 10.3390/s19020369

**Published:** 2019-01-17

**Authors:** Yufeng Fan, Xiaodong Zhu, Hulin Sui, Haotai Sun, Zhongming Wang

**Affiliations:** 1 Shenyang Fire Research Institute of Ministry of Public Security, ShenYang 110034, China; suihulin@syfri.cn; 2 School of Computer Science and Technology, Jilin University, Changchun 130025, China; s88500860@163.com; 3 Beijing Fubang Smart IoT Technology Co. Ltd, Haidian District, Beijing 100193, China; wangzhongming@fubangyun.com

**Keywords:** toxic and harmful gas monitoring, machine learning, weight model, fire command and dispatch

## Abstract

In recent years, fire accidents in petrochemical plant areas and dangerous goods storage ports in China have shown a trend of frequent occurrence. Toxic and harmful gases are diffused in the scenes of these accidents, which causes great difficulties for fire fighting and rescue operations of fire fighting forces, and consequently, casualties of firefighters often occur. In order to ensure the safety of firefighters in such places, this paper designs a monitoring system of toxic and harmful gases specially used in fire fighting and rescue sites of fire forces, and establishes the transmission network, monitoring terminal and data processing software of the monitoring system of toxic and harmful gases, establishing the danger model of the monitoring area of toxic and harmful gas-monitoring terminal, and the danger model of fire fighters’ working area, fusing the field toxic and harmful gas data, terminal positioning data, and field environmental data, designing the data structure of the input data set and the network structure of the RNN cyclic neural network model, and realizing the dynamic early warning of toxic and harmful gases on site.

## 1. Introduction 

In recent years, there has been a high incidence of fire accidents in petrochemical areas and dangerous chemical storage ports. In particular, in recent years, a number of major safety incidents have caused significant casualties and huge economic losses, which have caused great negative impact on society. Fire accidents in petrochemical areas and ports where hazardous chemicals are stored are typically characterized by the release of large quantities of toxic and harmful gases at the scene [1,2]. The presence of toxic and harmful gases has made it more difficult for fire fighters to carry out fire fighting and rescue in such scenes, and has also posed a great threat to the safety of the combatants at the scene. Therefore, the study of toxic and harmful gas monitoring systems is of great significance for early warning of fire and ensuring the life safety of field combatants.

With the rapid development of computer technology, modern control theory technology, sensors, and Internet of Things communication technology, various toxic and harmful gas monitoring technologies based on wireless communication networks are widely used in many industries, including petroleum, petrochemical, coal, municipal, fire, metallurgy, gas, medicine, telecommunications, electricity, and food processing [2,3]. A toxic gas monitoring system featuring intelligence, automation, and online features has also been developed [4,5,6,7,8]. In particular, the mature application of wireless communication technology provides a mature basic network support for the collection, detection, monitoring, and early warnings of toxic and harmful gases in special environments [9,10,11,12,13,14]. It also solves the “last mile” information transmission problem for toxic and harmful gas monitoring systems. It also makes it possible to explore wireless-based indoor and outdoor toxic and harmful gas monitoring and sensing systems in various industries [15,16,17,18]. Especially in underground mines, tunnels, and other places, the application is more advanced [19,20].

Relevant literature reports also indicate that within a closed space, the application that uses a wireless communication network to replace the traditional wired link to connect the gas sensors of various parts and transmits the data collected by the sensor to the monitoring center through the wireless network to construct a closed-space toxic and harmful gas monitoring and sensing system is relatively successful. Paik et al. [21], Pilsak et al. [22], and Zaharia et al. [23] respectively put forward the idea of using WiFi, ZigBee, and Bluetooth wireless communication technology to realize the construction of wireless network in hull space. Based on the above assumption, Kdouh et al. [24] and Park et al. [25] carried out a lot of communication performance tests in a hull environment. The conclusion is that the effect of wireless network coverage in metal enclosed space using the above high frequency and low power communication means is not so ideal. Then Carlos et al. [26] proposed using multi-hop self-organizing wireless communication network to solve the non-visual communication problem inside and outside the cabin, which provides a new solution for the application of toxic and harmful gas monitoring systems in ships and other closed spaces.

Petrochemical areas and dangerous chemical storage ports are usually in outdoor open space. The traditional ground-wired communication method cannot guarantee the effectiveness of the communication link in the event of a disaster accident. Therefore, most of the communication networks relying on the toxic and harmful gas monitoring system currently use the public mobile communication network [27]. However, in the actual application process, it is found that there are many problems in the public mobile communication network. The main reason is that the number of rescue personnel surges on scene or the power supply is disconnected at the time of the accident, which often leads to the congestion of public mobile communication network and it is difficult to guarantee communication stability. Therefore, deploying a wireless communication private network with strong penetration and wide coverage in such places is the only feasible technical solution.

Petrochemical areas and dangerous chemicals storage ports has brought great difficulties to fire fighting and rescue operations of fire fighting forces. How to effectively guarantee the safety of fire fighters in this environment has always been a major problem puzzling the fire forces. Existing toxic and harmful gas monitoring devices or systems are often unable to meet the actual needs of fire fighting and rescue field, and it is mainly reflected in the following aspects:

(1) The fire fighting unit has its own dedicated fire communication command system and a mobile communication command center (communication command vehicle) at the disaster accident site, and its mobile communication command center also deploys software and hardware systems dedicated to fire fighting forces. However, the existing toxic and harmful gas monitoring devices or systems are independently constructed and cannot be connected and integrated with the fire communication communication command system. They simply provide monitoring parameters for toxic and harmful gases, and it is difficult to provide a scientific basis for rescue command and dispatch at the scene;

(2) Although existing toxic and harmful gas monitoring devices or systems can have wireless remote transmission functions, their wireless transmission networks mostly use public mobile communication networks, and its stability and reliability of the network cannot be guaranteed at the disaster accident site. For example, the public mobile communication network at the scene of most disaster accidents may be paralyzed due to the occurrence of disaster accidents, and data communication cannot be transmitted [4,9,10];

(3) The existing monitoring devices for toxic and harmful gases can only detect the concentration of toxic and harmful gases in a given range, lacking a scientific trend development model. This leads to the fact that fire fighters can only judge how to command operations in the next stage based on the existing parameters or experience, which has little practical effect on operational command;

(4) The existing toxic and harmful gas monitoring devices do not possess explosion-proof characteristics. However, most of the toxic and harmful gases are flammable gases, so if the electronic equipment does not have explosion-proof characteristics: it is easy to become a source of ignition, resulting in site explosion and secondary disasters [7].

Based on the above analysis, this paper proposes to build a special monitoring system of toxic and harmful gases for the fire fighting and rescue field, using the temporary software and hardware system deployed at the scene to realize the monitoring of toxic and harmful gases in the fire fighting and rescue site, establishing the monitoring area danger model of the monitoring terminal of toxic and harmful gases, the danger model of the fire fighters in the working area at the scene, and training the data model by machine learning algorithm. The system can provide scientific decision support for commanders’ dispatching and commanding in fire fighting and rescue field, and guarantee the life safety of combatants at the scene.

## 2. System Design for Toxic and Harmful Gas Monitoring

**Figure 1:**
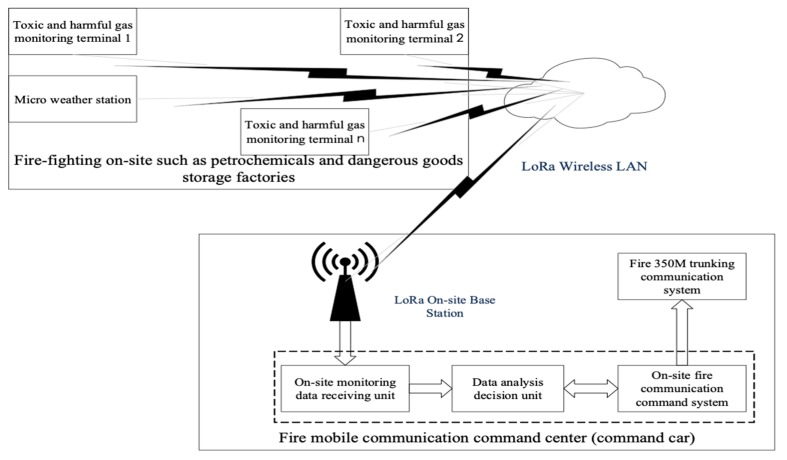
Schematic diagram of toxic and harmful gas monitoring system.

A monitoring system for toxic and harmful gases specially designed for fire fighting and rescue sites of fire fighting forces is designed in this paper. The system uses the terminal of toxic and harmful gas monitoring to collect the data of toxic and harmful gases at the disaster scene in real time, and then transmits the data to the fire mobile communication command center through the LoRa special communication network. Its mobile communication command system collects toxic and harmful gas data, terminal positioning data and field environment data, which can be used to generate a dangerous model of the monitoring area of toxic and harmful gas, monitoring terminal, and a dangerous model of fire fighters in the field operation area. The field commander dispatches fire fighters to carry out fire fighting, reinforcement, and evacuation commands in real time according to the output changes of the model, and carries out fire fighting and rescue operations on the premise of ensuring the life safety of fire fighters. The system schematic diagram is shown in Figure 1 below.

### 2.1. Schematic Diagram of Toxic and Harmful Gas Monitoring System

A monitoring system for toxic and harmful gases specially designed for fire fighting and rescue sites of fire fighting forces consists of: LoRa field base station (1 station) located in the fire mobile communication command center (command car), on-site monitoring data receiving unit, data analysis decision-making unit, on-site fire communication command system, fire fighting 350 M trunking communication system (located in the petrochemical industry), dangerous goods storage plant area, and other fire-ighting units at the fire and rescue site of a number of toxic and harmful gas monitoring terminals (not more than 256), micro weather station (1 station) and on-site LoRa wireless LAN. The on-site monitoring data receiving unit, the data analysis decision-making unit, and the on-site fire communication command system belong to the software function module, and are installed and deployed on the same computer in the command vehicle. The computer establishes a connection with the LoRa field base station through the RJ45 interface or the switch to exchange data. 

The LoRa field base station is deployed at the fire mobile communications command center (command car). The antenna is deployed on the roof of the command car and adopts a temporary deployment method (that is: when a fire accident occurs, the command car enters the scene and then sets up the base station antenna to power up the base station to normal operation with the communication coverage is generally up to a radius of 10 km). Only one LoRa base station needs to be opened at each site to establish an on-site LoRa wireless local area network, and the 433 MHz wireless LoRa signal covers the entire fire fighting and rescue site to realize the toxic and harmful gas monitoring terminal and the micro weather station to access the LoRa wireless local area network.

The on-site monitoring data receiving unit is connected with the LoRa site base station to receive the monitoring data which sent by several on-site toxic and harmful gas monitoring terminals and contains the toxic and harmful gas species, the gas concentration and the location data of the terminal, and then transmit the received data to the data analysis and decision-making unit.

The data analysis decision-making unit is connected with the field-monitoring data receiving unit to establish one-way connection, receiving data uploaded from the field-monitoring data receiving unit, establishing two-way connection with field fire fighting communication command system, adjusting and canceling the data of the Geographic Information System (GIS), environmental data (temperature, humidity, wind power, wind direction, air pressure), the combat position data of fire fighters, and warning parameters of toxic and harmful gases from the fire fighting communication command system, fusing the real-time monitoring data of the fire communication command system and the toxic and harmful gas, using the danger model of the monitoring area of toxic and harmful gas monitoring terminal and the danger model of fire fighters working area for fusion calculation, and uploading the model output to the fire communication command system in real time.

The field fire communication command system is deployed in the field mobile communication command center (command vehicle) to establish a two-way connection with the data analysis and decision-making unit, providing fire GIS data, field environment data (temperature, humidity, wind power, wind direction, air pressure), combat position data of fire fighters, and parameter data of toxic and harmful gas warnings for data analysis and the decision-making unit, receiving the danger model of the monitoring area of the toxic and harmful gas monitoring terminal and the output data of the danger model of the fire fighter’s working area on the spot, providing data support for on-site commanders to make scientific decisions, connecting with fire 350 M trunking communication system, and delivering the commander’s attack, reinforcements, and evacuation instructions to each combat fireman by voice.

The fire fighting unit 350 M trunking communication system is a point-to-point and point-to-multipoint cluster intercommunication means for the fire fighting forces of China. The system uses 12.5 kHz TDMA dual-slot, 4FSK modulation and digital voice compression technology, is mainly used for private network command and dispatch communication. The advantage lies in the rapid response and convenient application of large-area wireless communication, which can provide users with multi-level scheduling services. The on-site commander and the participating fire fighters use the handheld digital walkie-talkie for voice communication to realize on-site fire force deployment and personnel dispatching command. The fire fighting 350 M trunking communication system is deployed inside the on-site mobile communication command center (command vehicle), establishing a one-way connection with the on-site fire communication command system, and voicing the commander’s attack and evacuation commands to reach each combat fire fighter, so as to realize on-site voice dispatching command.

### 2.2. Schematic Diagram of Toxic and Harmful Gas Monitoring Terminal at fire fighting and Rescue Site

**Figure 2:**
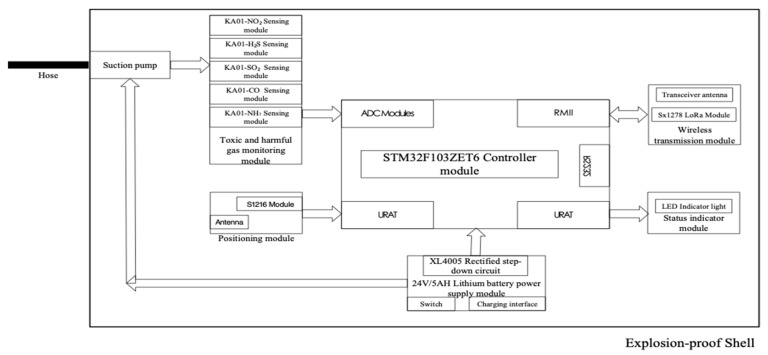
The Terminal Schematic.

The terminal for monitoring toxic and harmful gases in the fire fighting and rescue site is composed of controller module, wireless transmission module, status indicator module, multiple toxic and harmful gases monitoring module, positioning module, rectification and voltage reduction module, lithium battery power supply module, and explosion-proof shell. The on-site toxic and harmful gas monitoring terminal can collect the toxic and harmful gas types and concentration data in the field in real time, and transmit them to the on-site mobile communication command center through the on-site LoRa wireless base station in real time. The on-site toxic and harmful gas monitoring terminal data real-time collection cycle and data real-time upload cycle can be customized. In this paper, the data collection cycle and data upload cycle are both 10 s. The terminal schematic is shown in Figure 2 below:

The controller module uses the 32-bit ARM microcontroller STM32F103ZET6 from STMicroelectronics as the main control board, the 1 ADC Modules interface is connected with the toxic and harmful gas monitoring module to receive the toxic and harmful gas concentration data reported by the toxic and harmful gas sensing module in real time; the 1 RMII Ethernet interface is connected with the wireless transmission module, 1 RMII Ethernet interface is connected with the wireless transmission module, collecting the data by the terminal which is connected to the LoRa wireless local area network in the air through the wireless transmission module, and transmitting to the fire mobile communication command center in the field through the air interface connection; the 1 URAT interface is connected to the positioning module to receive positioning data of the terminal; the 1 URAT interface is connected with the status indication module to indicate the running status of the terminal through the LED indicator; the 1 RS232 interface is used to configure the internal parameters of the toxic and harmful gas monitoring terminal.

The toxic and harmful gas monitoring module is connected to the suction pump, including KA01-NH3 ammonia gas sensing module, KA01-CO carbon monoxide sensing module, KA01-SO_2_ sulfur dioxide sensing module, KA01-H_2_S hydrogen sulfide sensing module and KA01-NO_2_ nitrogen dioxide sensing module, collecting the simulated parameters of various toxic and harmful gases in the gas sent by the getter pump, connecting to the STM32F103ZET6 controller through the ADC Modules interface after AD conversion, and uploading monitoring data in real time.

The positioning module is composed of S1216 module and dual-mode antenna, supporting GPS + Beidou dual positioning, connecting with STM32F103ZET6 controller through URAT interface, and uploading the longitude, latitude and altitude positioning data of the terminal in real time.

The status indicator module is composed of four LED indicators, which are connected with STM32F103ZET6 controller through URAT interface. One LED indicator shows the connection status of terminal network, 1 LED indicator shows the working status of power supply, 1 LED indicator shows the working body of toxic and harmful gas monitoring module, and 1 LED indicator shows the working status of the positioning module.

The wireless transmission module is composed of Sx1278 LoRa module and receiving and receiving antenna, connecting with STM32F103ZET6 controller through RMII interface, realizing air access to the field LoRa wireless network, and transmitting the controller information to the field mobile communication command center.

The lithium battery power supply module is composed of a 24V5AH lithium battery, switch, charging port, and XL4005 rectifier step-down circuit, providing DC24V power supply support for the air suction pump, and DC3V power supply support for the terminal controller and other internal modules after rectification and step-down.

The suction pump is connected to the hose to send external gas samples into the toxic and harmful gas monitoring module, and can collect gas samples at a long distance by controlling the length of the hose, and the length of the hose does not exceed 20 m.

The shell of toxic and harmful gas monitoring terminal in fire fighting and rescue site is packaged with explosion-proof shell, which ensures that the terminal has explosion-proof characteristics and meets the safety requirements of fire fighting and rescue sites in petrochemical plant areas and dangerous chemicals storage ports.

## 3. Data Transmission and Processing Method for Toxic and Harmful Gas Monitoring System 

**Figure 3:**
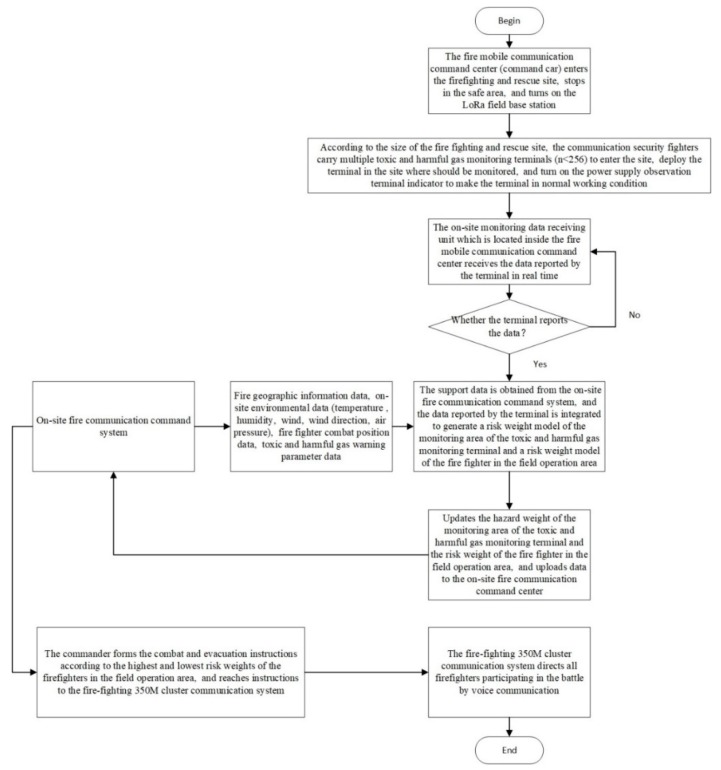
Data transmission processing flow chart.

A data transmission and processing method for a toxic and harmful gas monitoring system dedicated to the fire-ighting and rescue site of a fire-ighting unit is shown in Figure 3 below.

## 4. Hazard Model of the Monitoring Area of the Toxic and Harmful Gas Monitoring Terminal and Hazard Model of the Fire Fighter in the Field Operation Area 

The smoke diffusion model is a physical or mathematical model describing the transfer, diffusion, and dilution of atmospheric environment to gas diffusion sources and is also one of the powerful means of disaster prediction, personnel support, and rescue command for accident rescue forces. The main factors affecting the diffusion of flue gas include the leakage rate of the diffuser, the height of the diffuser, the initial concentration, the diffusion characteristics of different gases, the environmental information of the site and the leakage time [28]. The main modeling method used in the smoke diffusion model is the Gauss smoke diffusion model, which predicts the smoke concentration at a certain location at the next moment based on the diffusion parameters [29]. The Gauss smoke diffusion model belongs to the laboratory theoretical modeling model. The premise is that all diffusion parameters can be obtained, but in the accident scene, many diffusion parameters can not be obtained in real time, such as the location, leakage, and height of the diffusion source, so the predicted flue gas concentration is not very accurate in accuracy and persistence. However, the Gauss smoke cluster expansion model plays an important role in the post-accident simulation and deduction, and it plays an important role in dealing with disaster accidents in similar scenarios. However, the Gauss smoke mass diffusion model plays an important role in the simulation deduction after the accident and in the subsequent disaster scenes of similar scenes.

The emergence of machine learning provides a new solution for the prediction of smoke diffusion models. Machine Learning (ML), as a multi-disciplinary interdisciplinary subject, involving probability theory, statistics, approximation theory, convex analysis, algorithmic complexity theory, and other disciplines, is also a core discipline of Artificial Intelligence (AI) [30], and mainly studies the use of computer simulation or the realization of human learning behavior, to reorganize the existing knowledge structure so as to continuously improve its performance. From the point of view of the realization method of machine learning, machine learning model is more inclined to a “black box” processing process, and researchers can not use the existing theoretical system to deduce and verify the algorithm of machine learning logically. However, the existing achievements of machine learning, especially in image classification, image recognition, speech recognition and other fields, show that machine learning can solve some complex and computational tasks.

The basic idea of machine learning for data manipulation and data processing can be summarized as follows:

(1) Collecting input data and form data matrix as input layer;
(2) The hidden layer makes multiple convolutions of the data to extract the data characteristics;
(3) The output layer performs binding output on the hidden layer processed vector data;
(4) Selecting the appropriate loss function to evaluate the processing results;
(5) Comparing the input data labels to assess whether the model is appropriate.

The above process is described by the formula as follows:

(1) y=wX+b

In the above formula, *y* represents the output data range and can also represent the category of the output, *X* represents the input data, *w* represents the weight of the input data, and b represents the data offset. The model training process ultimately needs to determine the appropriate *w* and *b* parameters for predicting subsequent actual input data.

Different from image recognition and classification tasks in machine learning tasks, the input data in this paper are collected by various field sensors with time characteristics. Therefore, the neural network used in this paper is RNN (recurrent neural network). Time series data refers to the data collected at different time points, which reflects the state or degree of change of something, phenomenon and so on over time, and RNN is a kind of neural network specially used to process time series data [31].

**Figure 4:**
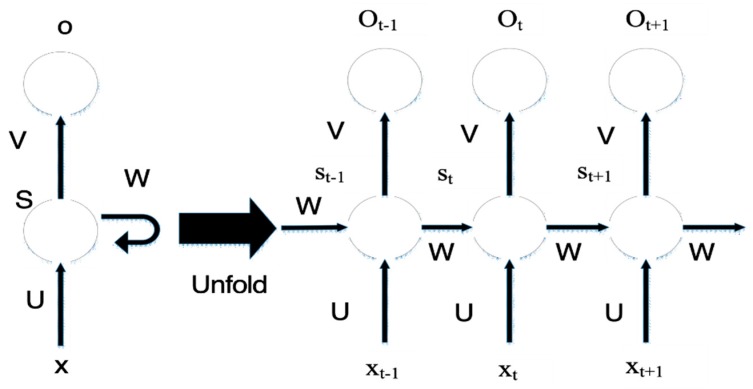
RNN (recurrent neural network) Network Architecture.

A typical RNN network structure is shown in Figure 4 below.

The left side of the figure shows the working principle of the RNN network structure, and the right side shows the expansion of the left structure, which x represents the input layer, o represents the output layer, s represents the hidden layer, t refers to the calculation of times, and V,W,U is the weight. When calculating the tth times hidden layer state, it can be expressed as $S_{t}=\left(U\times X_{t}+W\times S_{\left(t-1\right)}\right)$.

### 4.1. Input Parameter

In this paper, two models of danger in monitoring area of on-site toxic and harmful gas monitoring terminal and that of fire fighters in on-site operations area are established. The danger model of monitoring area of field toxic and harmful gas monitoring terminal is used to characterize the danger of monitoring terminal location, which is quantified by numbers. The danger model of fire fighter working area is used to characterize the danger of fire fighter working area, which is also quantified by numbers.

The input data for both models contains the following:

(1) Fire fighting and rescue site environment, including: temperature, humidity, air pressure, wind direction, wind;

(2) The data collected by the toxic and harmful gas monitoring terminal at the fire and rescue site, including the geographical location, the type and concentration of toxic and harmful gases;

(3) The data and geographical location of the fire fighters participating in the fire fighting and rescue site;

(4) The “chemical dangerous goods disaster accident disposal plan system” currently used by the fire brigade can provide explosion parameters, low limit alarm concentration, high limit alarm concentration, gases’ lethal concentration, and different kinds of toxic and harmful gases on site. 

### 4.2. Model Principle 

(1) On-site toxic and harmful gas monitoring terminal monitoring area risk model

Assuming that A is used as the output of regional hazard model for monitoring toxic and harmful gases, the factors affecting the output of the model should include: real-time environmental parameters (temperature, humidity, air pressure, wind direction, wind force) in the field, which represented by E; types and concentrations of toxic and harmful gases collected by the field terminal, which represented by G. The types of toxic and harmful gases, explosion parameters, low alarm concentration, high alarm concentration, gases’ lethal concentration, gas severe stimulation concentration, and real-time gas concentration static data can be used as input data labels, and quantified to evaluate the fitness of the model.

It is concluded that the regional risk model for monitoring terminal sites of toxic and harmful gases is as follows:

(2) $$A_{i}\left(t\right)=\overset{n}{\underset{j=0}{\sum }}\left(E\left(t\right)+G_{j}\left(t\right)\right)$$

I represents the monitoring terminals of toxic and harmful gases at different locations on site, T represents time, and j represents different kinds of toxic and harmful gases.

(2) Hazard model for fire fighters in field operation area

Taking B as the risk model output of fire fighters in the field operation area, the main factors affecting the model output are the output value of the danger model of the monitoring area of the field toxic and harmful gas monitoring terminal and whether the fighters are within the measurement range of a toxic and harmful gas monitoring terminal.

It is concluded that hazard models of fire fighters in the field area are as follows:

(3) $$B_{k}(t)=\underset{0\leq i\leq n}{max}(A_{i}(t)\times D)$$

In the above formula, A represents the danger model of the monitoring area of the poisonous and harmful gas monitoring terminal; D represents whether the fire fighter is within the measuring range of a poisonous and harmful gas monitoring terminal and takes the value within the effective measuring range of 1, otherwise 0; K represents a fire fighter on the spot; n represents the number of the monitoring terminal of the poisonous and harmful gas on the scene.

### 4.3. RNN Model Construction

(1) Structure Design of Model Training Data Set

The input parameters included in the two models of the hazard weights of the monitoring area of the field toxic and harmful gas monitoring terminal and the hazard weights of the fire fighters in the field operation area are described in detail in Section 4.1. In this section, all parameters need to be numeralized and the structure of training data set is established.

The input parameters of the hazard weights of the monitoring area in the field toxic and harmful gas monitoring terminal should include the field environmental data and the field toxic and harmful gas data. Field environmental information includes temperature, humidity, air pressure, wind direction, and wind force, where wind direction data is text data. We use one-hot encoding to extract text features. The resulting encoding is shown in Table 1 below.

The main purpose of this paper is to monitor the toxic and harmful gas types and concentration changes at the terminal according to the monitoring of toxic and harmful gases at the fire fighting and rescue site, combining the data with the influence of on-site environmental factors, predict the potential dangers of toxic and harmful gases in the follow-up events to the fire fighters on the scene, and then give early warning. Therefore, it is necessary to define the concentration of toxic and harmful gases in the field according to the scientific toxic concentration injury standard, which states in what concentration range the personnel must evacuate immediately and take protective measures and in what concentration range the personnel only need to take local protective measures, in order to minimize the immediate and effective harm of toxic gases to the field personnel. At present, the Emergency Response Planning Guidelines (ERPGs) [32] formulated by the American Industrial Health Association (AIHA) have been widely used, which specify three concentrations (ERPG-1, 2, 3) to describe exposure. ERPG-1 represents the mild injury area, the time limit of toxic load in the mild injury area is 60 min; ERPG-2 represents the severe injury area, the time limit of toxic load in the severe injury area is 30 min; ERPG-3 represents the lethal area, and the time limit of toxic load in the lethal area is 30 min. We expanded the concentration of ERPG classification, and increased the two classifications of ERPG-0, which represent the harmless zone and the harmless zone, whose load time limit is infinite.

The toxic and harmful gas monitoring terminal monitoring area risk model output is ERPG-1, ERPG-2 and ERPG-3, and we also perform one-hot coding for extracting their characteristics, and the coding is shown in Table 2 below:

The structure of the final design model training data set is shown in Table 3 below:

In the table above, $t_{n}$ represents the data sampling at that time and P represents the value.

(2) RNN Model Structural Design

**Figure 5:**
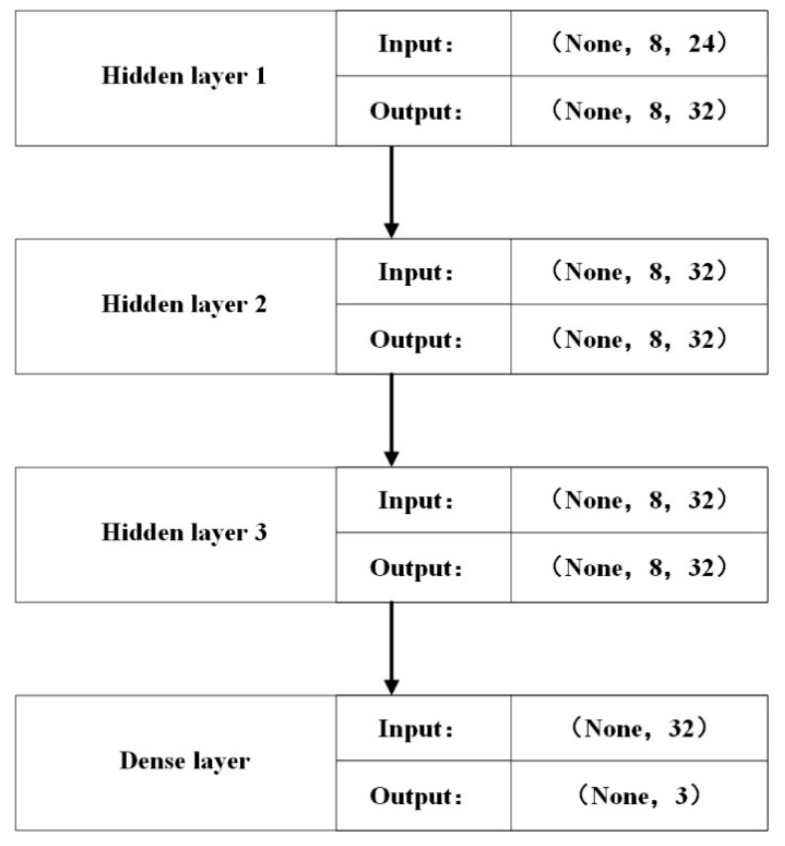
RNN model structure.

The RNN model structure designed in this paper is shown in Figure 5 as follows:

The RNN model in the figure above consists of three hidden layers and one neural network layer, in which the connection between hidden layers is fully connected to ensure the sharing of parameter weights, and the neural network layer is used to reduce the dimension of the operation results to three classifications. The activation function between hidden layers is Tanh activation function, and the activation function of the last layer of neural network is Relu activation function (the rectified linear unit) for output of hidden layer neurons. The weight parameters are updated iteratively using RMSprop algorithm. RMSProp has also proved to be an effective and practical algorithm for deep learning network optimization [33].

We introduce a mean square error loss function in method of calculation errors. We have established nth times environmental parameter samples $E_{i}$ and toxic and harmful gas parameters $G_{i}$. According to the calculation method of the mean square error loss function, we can derive the toxic and harmful gas monitoring terminal. The expression for monitoring the regional hazard model output $A_{i}$ is as follows:

(4) $$\mathrm{Los}\;(A_{i})=\frac{1}{n}\sum_{i=1}^{n}{\left(A_{i}-E_{i}-G_{i}\right)}^{2}$$

The above formula represents the hazard model of the monitoring area of the toxic and harmful gas monitoring terminal, n represents the number of environmental parameter samples and toxic and harmful gas parameters, $E_{i}$ represents the ith environmental parameter sample, $G_{i}$ represents the ith toxic and harmful gas parameter, and Los ($A_{i}$) represents toxic. The harmful gas monitoring terminal monitors the loss function of the regional hazard model.

Using the above loss function, we can get the deviation between the predicted value and the actual value. The smaller the loss value, the closer the predicted value is to the real value.

If the prediction is completely accurate, the above loss value should be 0. The more precise the data sample n is, the more real the loss value will be. At the same time, in practical application, we can also use the actual measured data as parameters to introduce the above model and calculate the loss value, and use the actual known parameters to correct and save all training data. In the specific implementation process, we can measure the actual operation parameters on the spot, import the actual operation parameters into the weight model, and then precisely match the training data to get the danger model output of all fighters on the spot. Commanders decide whether to issue withdrawal instructions according to ERPG classification standards to ensure the life safety of fighters on the scene.

## 5. Conclusions

Compared with the existing traditional toxic and harmful gas monitoring system, the toxic and harmful gas monitoring system and data transmission processing method designed in this paper for the fire fighting and rescue site of the fire brigade has the following improvements:

(1) System data acquisition application LoRa wireless communication method. Compared with the fixed deployment mode of the on-site collection network and the use of the public mobile communication network, this method has the characteristics of more secure and stable network. Moreover, the network in this way adopts a temporary deployment application mode, and has the characteristics of rapid and convenient network deployment. Because the occurrence of fire accidents is contingent and sudden, the LoRa wireless communication method is more targeted;

(2) The system and the fire brigade’s existing communication command system have achieved efficient integration. The existing toxic and harmful gas monitoring systems at the fire-ighting and rescue site are mostly closed systems, and the system does not exchange data with the outside, which makes the field commander more inconvenient when applying the system. The on-site harmful gas monitoring system designed in this paper can establish a connection with the fire brigade’s existing communication command system, which improves the commanding effectiveness of the commander;

(3) This paper introduces the machine learning method into the toxic and harmful gas monitoring system at the fire fighting and rescue site, integrating the data of toxic and harmful gases, terminal positioning data, and field environment data, designing the data structure of input data set and the network structure of RNN cycle neural network model, and establishing the regional danger model of monitoring terminal of toxic and harmful gases in the field of fire fighting. Real-time monitoring, trend prediction, and real-time warning of toxic and harmful gases at the site have been realized, which plays an important role in ensuring the safety of fire fighters participating in the battle;

(4) The toxic and harmful gas monitoring terminal designed in this paper has explosion-proof characteristics and reaches IP66 protection level. It has good adaptability in fire fighting, and in fire fighting and rescue sites such as petrochemical plant areas and hazardous chemicals storage ports.

## Figures and Tables

**Table 1:** Wind direction one-hot coding table.

Wind Direction	One-Hot Coding
East wind	[0, 0, 0, 0, 0, 0, 0, 1]
South wind	[0, 0, 0, 0, 0, 0, 1, 0]
West wind	[0, 0, 0, 0, 0, 1, 0, 0]
North wind	[0, 0, 0, 0, 1, 0, 0, 0]
Southeast wind	[0, 0, 0, 1, 0, 0, 0, 0]
Northeast wind	[0, 0, 1, 0, 0, 0, 0, 0]
Southwest wind	[0, 1, 0, 0, 0, 0, 0, 0]
Northwest wind	[1, 0, 0, 0, 0, 0, 0, 0]

**Table 2:** Model output weight one-hot code table.

Model Output	One-Hot Code
ERPG-0	[0, 0, 0, 1]
ERPG-1	[0, 0, 1, 0]
ERPG-2	[0, 1, 0, 0]
ERPG-3	[1, 0, 0, 0]

**Table 3:** Model training data set data structure table.

	Model Input X										Model Output Y
Time Series	Temperature	Humidity	Pressure	Wind Direction	Wind Power	Concentration					ERPG Value
						NO_2_	H_2_S	SO_2_	CO	NH3	
$t_{1}$	[p]	[p]	[p]	[p]	[p]	[p]	[p]	[p]	[p]	[p]	[p]
$t_{2}$	[p]	[p]	[p]	[p]	[p]	[p]	[p]	[p]	[p]	[p]	[p]
$t_{3}$	[p]	[p]	[p]	[p]	[p]	[p]	[p]	[p]	[p]	[p]	[p]
…	…	…	…	…	…	…	…	…	…	…	…
$t_{n}$	[p]	[p]	[p]	[p]	[p]	[p]	[p]	[p]	[p]	[p]	[p]
